# Developmental Alterations in Motor Coordination and Medium Spiny Neuron Markers in Mice Lacking PGC-1α

**DOI:** 10.1371/journal.pone.0042878

**Published:** 2012-08-14

**Authors:** Elizabeth K. Lucas, Sarah E. Dougherty, Laura J. McMeekin, Alisa T. Trinh, Courtney S. Reid, Rita M. Cowell

**Affiliations:** 1 Department of Neuroscience, Mount Sinai School of Medicine, New York, New York, United States of America; 2 Department of Psychiatry & Behavioral Neurobiology, University of Alabama at Birmingham, Birmingham, Alabama, United States of America; Claremont Colleges, United States of America

## Abstract

Accumulating evidence implicates the transcriptional coactivator peroxisome proliferator activated receptor γ coactivator 1α (PGC-1α) in the pathophysiology of Huntington Disease (HD). Adult PGC-1α ^−/−^ mice exhibit striatal neurodegeneration, and reductions in the expression of PGC-1α have been observed in striatum and muscle of HD patients as well as in animal models of the disease. However, it is unknown whether decreased expression of PGC-1α alone is sufficient to lead to the motor phenotype and striatal pathology characteristic of HD. For the first time, we show that young PGC-1α ^−/−^ mice exhibit severe rotarod deficits, decreased rearing behavior, and increased occurrence of tremor in addition to the previously described hindlimb clasping. Motor impairment and striatal vacuolation are apparent in PGC-1α ^−/−^ mice by four weeks of age and do not improve or decline by twelve weeks of age. The behavioral and pathological phenotype of PGC-1α ^−/−^ mice can be completely recapitulated by conditional nervous system deletion of PGC-1α, indicating that peripheral effects are not responsible for the observed abnormalities. Evaluation of the transcriptional profile of PGC-1α ^−/−^ striatal neuron populations and comparison to striatal neuron profiles of R6/2 HD mice revealed that PGC-1α deficiency alone is not sufficient to cause the transcriptional changes observed in this HD mouse model. In contrast to R6/2 HD mice, PGC-1α ^−/−^ mice show increases in the expression of medium spiny neuron (MSN) markers with age, suggesting that the observed behavioral and structural abnormalities are not primarily due to MSN loss, the defining pathological feature of HD. These results indicate that PGC-1α is required for the proper development of motor circuitry and transcriptional homeostasis in MSNs and that developmental disruption of PGC-1α leads to long-term alterations in motor functioning.

## Introduction

Huntington disease (HD) is a debilitating autosomal dominant disorder involving progressive deterioration of motor and psychiatric functioning over a period of years, leading to death. HD is caused by an expansion of CAG repeats in the huntingtin gene [1]. A hallmark in the pathology of HD is severe atrophy of the striatum mediated by a preferential loss of the medium spiny projection neurons (MSNs) in this region [2], although other brain regions and cell types are also affected to a lesser extent by the presence of mutant huntingtin (mHtt) [3], [4]. Over time, mHtt aggregates to form cytosolic and intranuclear inclusions. It is currently unknown if loss of function of native huntingtin protein and/or gain of function of the mutant protein results in the cell death observed in HD. However, there is a large body of evidence to support the involvement of mitochondrial dysfunction [5], [6] and transcriptional dysregulation [7] in HD pathology. Recently, the transcriptional coactivator peroxisome proliferator activated receptor γ coactivator 1α (PPARGC1A or PGC-1α) has been proposed to be a link between these features [8], [9].

Since its discovery in 1998, PGC-1α has been coined the “master regulator of metabolism” due to its ability to initiate transcription of genes involved in mitochondrial biogenesis and antioxidant defense in peripheral tissues and neurons [10]–[12]. Interest in the study of PGC-1α in the context of neurodegeneration was elicited by the initial report demonstrating that adult PGC-1α ^−/−^ mice exhibit hindlimb clasping and hyperactivity, behavioral abnormalities indicative of neurological dysfunction, accompanied by spongiform vacuoles predominately localized to the striatum [13]. Interestingly, PGC-1α transcript expression is dramatically upregulated in the rodent striatum during the early postnatal period [14], suggesting an early requirement for PGC-1α in this brain region. Thus, we hypothesized that the degenerative and behavioral phenotype of PGC-1α ^−/−^ mice may occur earlier than previously reported.

A growing body of evidence strongly implicates a role for PGC-1α in the pathophysiology of HD. Reductions in the expression of PGC-1α have been consistently observed in the striatum and muscle of patients with HD [15]–[18], potentially due to pathogenic interactions of mHtt with the PGC-1α promoter [15]. Polymorphisms in the PGC-1α gene have also been shown to affect the age of onset of HD [19]–[21]. Furthermore, reduced PGC-1α expression has also been reported in striatal homogenates from various animal models of HD, although reductions in peripheral tissues such as muscle are generally much greater than those found in the brain [16], [17], [22], [23]. Interestingly, using cell-specific laser capture microdissection and mRNA analysis, Cui et al. (2006) demonstrated selective loss of PGC-1α in MSNs in a mouse model of HD [15]. It is unclear, however, if loss of PGC-1α leads to the MSN degeneration and progressive motor phenotype characteristic of this disease.

In order to investigate the involvement of PGC-1α in the progression of motor abnormalities and striatal pathology more precisely, we conducted a battery of behavioral and transcriptional assays in PGC-1α ^+/+^, ^+/−^, and ^−/−^ littermates at four and twelve weeks of age. We hypothesized that if a loss of PGC-1α leads to the transcriptional changes and reduced cellular viability observed in HD, then the motor phenotype and cellular target(s) of striatal degeneration in PGC-1α ^−/−^ mice would closely mirror those observed in animal models of HD. Here, we utilize the well-characterized R6/2 mouse model of HD, which carries transgenic expression of exon 1 of the human huntingtin gene with an expanded CAG repeat [24]. While this mouse model of HD is quite severe and models juvenile HD more closely than adult-onset HD, it is the most appropriate model to compare to PGC-1α ^−/−^ mice when considering the early onset of neurodegeneration and motor symptoms that we observed in PGC-1α ^−/−^ mice.

We show that mice lacking PGC-1α exhibit striatal spongiform vacuoles and severe behavioral abnormalities by four weeks of age, before the previously published onset of symptoms in the R6/2 HD mouse model [25]. Neurodegeneration and motor deficits are completely recapitulated by conditional nervous system deletion of PGC-1α and do not progressively worsen between four and twelve weeks of age. Transcriptional profiling of MSN and interneuron markers between mice lacking PGC-1α and R6/2 HD mice reveal that gene expression changes do not mirror, but often directly oppose, one another. In fact, almost all MSN markers are increased in PGC-1α ^−/−^ mice at twelve weeks of age, a time when almost all tested transcripts are decreased in the R6/2 HD mouse. This study highlights the differences between the PGC-1α ^−/−^ mice and the R6/2 mouse model of HD with respect to pre-adolescent and adult changes in the transcriptional profiles of striatal neuronal populations. Furthermore, these studies indicate that reductions in PGC-1α expression are associated with dramatic transcriptional compensatory changes within MSNs, suggesting that this cellular population is dependent on PGC-1α for transcriptional homeostasis.

## Materials and Methods

### Animals

All experimental protocols were approved by the Institutional Animal Care and Use Committee of the University of Alabama at Birmingham. PGC-1α ^−/−^ mice [13] were used for experiments, and PGC-1α ^+/+^, ^+/−^, and ^−/−^ mice were obtained from offspring of PGC-1α ^+/−^ breeding pairs maintained on a C57BL6/J genetic background (gift of Jiandie Lin, University of Michigan) and genotyped according to the primer sets published in Lin et al. (2004). Conditional nervous system deletion of PGC-1α was achieved by crossing mice with loxP sites flanking a critical exons of the PGC-1α gene ([13]; gift of Bruce Spiegelman, Harvard Medical School) with mice expressing cre recombinase driven by the nestin promoter (gift of Albert LaSpada, University of California, San Diego) maintained on a C57BL6/J genetic background. LoxP sites were detected by the forward primer TCCAGTAGGCAGAGATTTATGAC and the reverse primer TGTCTGGTTTGACAATCTGCTAGGTC, and cre recombinase was detected by the forward primer CCGGGCTGCCACGACCAA and the reverse primer GGCGCGGCAACACCATTTTT. R6/2 mice [24] carrying exon 1 of the human huntingtin gene with 160±10 CAG repeats were maintained on a hybrid B6CBA genetic background (purchased from Jackson Laboratories, Bar Harbor, ME, USA, #002810) and were genotyped according to the specifications in [24]. All mice were housed two to five in a cage at 26±2°C room temperature with food and water ad libitum.

### Behavioral analyses

Behavioral analyses were conducted on littermates at four and twelve weeks of age in a repeated-measures design. All experiments were conducted during the lights-on period (6am–6pm), and experimenters were blind as to genotype of the animals.

#### Rotarod

The rotarod apparatus (MedAssociates, St. Albins, VT, USA) consisted of a five-station treadmill with a computer-controlled stepper motor-driven drum with constant speed or accelerating speed modes of operation. Animals were trained on the rotarod for four days. During the training period, animals were placed on the treadmill at a constant speed of 24 rotations per minute (rpm) for a maximum of 60 seconds for a total of four trials per day. On the fifth day, animals underwent two trials each at rotating speeds of 0–10 accelerating and 16, 20, 24, 28, and 32 fixed rpm. Each trial lasted for a maximum of 60 seconds, during which latency to fall was recorded. Mice were allowed to rest for at least five minutes between each trial.

#### Qualitative behavior

On rotarod test day, mice were assessed for the presence or absence of hindlimb clasping and tremor. Tremor was assessed by placing each mouse in a holding cage for observation over a 60-second period. If the trunk of the body was clearly shaking while the mouse was immobile, the mouse was coded as exhibiting a tremor. Hindlimb clasping was assessed by suspending the mouse by its tail for 15 seconds. If at any time during the 15-second suspension the mouse clasped the two hindlimbs together or clasped a forelimb to a hindlimb, the mouse was coded as exhibiting hindlimb clasping.

#### Open field

Animals were placed in a square apparatus (27.9 cm^2^) consisting of 48 infrared beams (MedAssociates) in a dark room for 30 minutes. Data were collected with Open Field Activity Software (MedAssociates) in one-minute intervals over the test period. Parameters of interest collected from the software included ambulatory distance, ambulatory time, and number of rears.

### Gene expression analyses

Mice were anesthetized with isoflurane before sacrifice by decapitation. Brains were rapidly removed, and striata were dissected by gross anatomical markers. For conditional knockouts, liver and mixed-type muscle from quadriceps femoris were also removed. Tissue pieces were collected in centrifuge tubes, flash frozen on dry ice, and stored at −80°C. Before processing, samples were incubated in RNA*later*®-ICE (Ambion, Austin, TX, USA) according to manufacturer's instructions. Tissue was homogenized with Tissue-Tearor (Biospec, Bartlesville, OK, USA) in Trizol reagent, and RNA was isolated by the Trizol/choloform-isopropanol method following the manufacturer's instructions (Invitrogen, Carlsbad, CA, USA). RNA concentrations and purity were quantified using a Thermo Scientific NanoDrop2000 (Fisher Scientific, Pittsburg, PA, USA). Equivalent amounts of RNA (1 µg) were treated with DNase I (Promega, Madison, WI, USA) at 37°C for 30 minutes, and DNase was inactivated at 65°C for 15 minutes. RNA was reverse-transcribed using the High-Capacity cDNA Archive Kit (Applied Biosystems, Carlsbad, CA, USA). Taqman q-RT-PCR was performed with JumpStart Taq Readymix (Sigma, St. Louis, MO, USA) and the mouse-specific Applied Biosystems primers listed in Table 1.

**Table 1:** List of Applied Biosystems primers with catalogue numbers.

	Transcript	Protein	Catalogue #
*General Projections*	Gad1	Glutamic Acid Decarboxylase 67	Mm00725661_s1
	Calb1	Calbindin	Mm00486645_m1
	Oprm	μ Opioid Receptor	Mm01188089_m1
*Direct Pathway*	Pdyn	Dynorphin	Mm00457572_m1
	Tac1	Substance P	Mm01166996_m1
	Drd1a	Dopamine Receptor 1a	Mm01353211_m1
*Indirect Pathway*	Penk1	Enkephalin	Mm01212875_m1
	Drd2	Dopamine Receptor 2	Mm00438541_m1
*Interneurons*	Pvalb	Parvalbumi	Mm00443100_m1
	Calb2	Calretinin	Mm00801461_m1
	NPY	Neuropeptide Y	Mm01212875_m1
	Chat	Choline Aceyltransferase	Mm01212875_m1
	Nos1	Neuronal Nitric Oxide Synthase	Mm00435175_m1
*PGC-1α*	Ppargc1a	Peroxisome Proliferator Activated Receptor γ Coactivator 1α	Mm00447183_m1
*Control*	Actb	β-Actin	Mm00607939_s1
	18S	18S ribosomal RNA	Hs99999901_s1

Reaction protocols consisted of an initial ramp cycle (2 minutes, 50°C; 10 minutes, 95°C) and 40 subsequent cycles (15 seconds, 95°C; 1 minute, 60°C). Relative concentrations of the genes of interest were calculated in comparison to a standard curve calculated from dilutions of cDNA (1∶5, 1∶10, 1∶20; calibrator method) from a pooled sample of wildtype littermate controls. For striatal data, values were normalized to β-actin values of the same sample, then expressed as arbitrary units (a.u.) ± SEM. Importantly, as β-actin was used for the striatal internal control throughout this study, mean β-actin levels did not significantly differ between PGC-1α ^−/−^, ^+/−^, and ^+/+^ animals at four weeks (^−/−^ = 1.38±0.10 a.u.; ^+/−^ = 1.13±0.10 a.u.; ^+/+^ = 1.17±0.17 a.u.) or twelve weeks (^−/−^ = 1.26±0.12 a.u.; ^+/−^ = 1.33±0.11 a.u.; ^+/+^ = 1.26±0.10 a.u.) of age. Mean actin values for R6/2 HD mice also did not significantly differ from WT mice at four weeks (R6/2 = 1.01±0.36 a.u.; WT = 1.35±0.35 a.u.) or twelve weeks (R6/2 = 1.04±0.07 a.u.; WT = 1.41±0.31 a.u.) of age. In peripheral tissues, however, we observed a significant difference between genotypes in β-actin expression; thus, 18S was used as the internal control for liver and muscle samples.

### Histology

Animals were anesthetized with isoflurane and perfused intracardially with phosphate-buffered saline (PBS, pH 7.4) and 4% paraformaldehyde in PBS. Brains were removed, postfixed in 4% paraformaldehyde for 24–72 hours, cryoprotected in graded sucrose (5–20%), embedded in a mixture of 20% sucrose and Tissue-Tek O.C.T. Compound (Sakura Finetek, Torrance, CA, USA), and frozen at −80°C. Tissue blocks were sectioned at 20 µm, mounted onto charged slides (Fisher, Hampton, NH, USA), and allowed to dry overnight before freezing at −80°C. For general histological analysis, Hematoxylin and Eosin (H&E; Sigma) staining was conducted according to the manufacturer's instructions.

For immunohistochemistry, primary antibodies included mouse monoclonal anti-GAD67 (Millipore, Billerica, MA, USA), mouse monoclonal anti-calbindin (Sigma), mouse monoclonal anti-parvalbumin (Sigma), rabbit polyclonal anti-NPY (Sigma), and rabbit polyclonal anti-tyrosine hydroxylase (Sigma). Sections were blocked with 10% serum from the host of the secondary antibody in PBS for 1 hour. Optimal staining of GAD67, parvalbumin, and tyrosine hydroxylase required antigen retrieval by incubation in citrate buffer (10 mM citric acid, pH 6.0; 37°C for 10 minutes, room temperature for 20 minutes, wash in PBS). Sections were then incubated in the primary antibody overnight with 5% serum from the host of the secondary antibody in PBS with Triton-X100 (PBST; Sigma) at 4°C.

For immunofluorescence staining (GAD67, calbindin, parvalbumin, and NPY), slides were washed in PBST and PBS and incubated for 2 hours at room temperature with the corresponding fluorescence-conjugated secondary antibodies with 5% serum and 3% BSA in PBST. After washes in PBST and PBS, sections were mounted with Prolong Gold Antifade with DAPI (Invitrogen). When necessary, a Vector Mouse on Mouse Kit was used to minimize background staining for mouse-made antibodies. Images were captured with a Leica confocal microscope. Settings for images collected on the confocal microscope were done by adjusting collection settings first (gain and offset) for genotypes expected to set the highest fluorescence intensity. Settings were then held constant across collected images for a given mouse line. This procedure was maintained for adjusted values using Adobe Photoshop CS3.

For diaminobenzamide staining (tyrosine hydroxylase), slides were washed in PBST and PBS and incubated for 1 hour at room temperature with the corresponding secondary antibody with 5% serum from the host of the secondary antibody in PBST. Slides were washed in PBST, peroxidase-quenched with 0.3% hydrogen peroxide in methanol, and incubated with horseradish-peroxidase labeled avidin-biotin complex (Vector Laboratories, Burlingame, CA, USA) for 30 minutes. After washes in PBS, antibody binding was visualized with nickel-enhanced diaminobenzamide (Vector Laboratories), and sections were dehydrated in graded ethanols and xylenes and mounted with nonaqueous mounting media. Selected images were captured using a SPOT camera and software system (Diagnostic Instruments, Sterling Heights, MI, USA) attached to a Leica DM5000B microscope (Leica, Bannockburn, IL, USA), and images were imported into Adobe Photoshop CS3 (Adobe, San Jose, CA, USA) for adjustments to contrast and brightness.

### High performance liquid chromatography (HPLC)

Animals were sacrificed using an *in vivo* Muromachi Microwave Fixation System (Stoelting Company, Wood Dale, IL, USA) to prevent enzymatic degradation of biogenic amines. Brains were removed, and striata were dissected by gross anatomical markers and sent to the CMN/KC Neurochemistry Core at Vanderbilt University for analyses.

Striata were homogenized in 100–750 µl of 0.1 M TCA, which contains 10^−2^ M sodium acetate, 10^−4^ M EDTA, 5 ng/ml isoproterenol (as internal standard) and 10.5% methanol (pH 3.8). The supernatant was separated by centrifugation, removed, and stored at −80°C until use. Biogenic amines were determined by a specific HPLC assay utilizing an Antec DECADE II electrochemical detector (DataApex, Prague, Czech Republic) operated at 33°C with an oxidation of 0.4. Twenty µl samples of the supernatant were injected using a Waters 717+ Autosampler (Meadows Instrumentation Inc., Bristol, WI, USA) onto a Phenomenex Nucleosil C18 HPLC column (150×4.60 mm; Torrance, CA, USA). Biogenic amines were eluted with a mobile phase consisting of 89.5% 0.1 M TCA, 10^−2^ M sodium acetate, 10^−4^ M EDTA and 10.5% methanol (pH 3.8). Solvent was delivered at 0.6 ml/minute using a Waters 515 HPLC pump (Meadows Instrumentation). Using this HPLC solvent, biogenic amines elute in the following order: noradrenaline, 3-Methoxy-4-hydroxyphenylglycol (MHPG), adrenaline, 3,4-Dihydroxyphenylacetic acid (DOPAC), Dopamine, 5-Hydroxyindoleacetic acid (5-HIAA/HVA), 5-hydroxytryptamine (5-HT), and 3-methoxytyramine (3-MT). HPLC control and data acquisition were managed by Empower software (Orlando, FL, USA).

### Statistical Analyses

All statistical analyses were conducted with SPSS software (IBM, Armonk, NY, USA). For behavioral analyses, repeated-measures ANOVA followed by planned comparisons with Holm-Bonferroni (rotarod), one-way ANOVA followed by Fisher's LSD (open field), or chi-square tests for independence (qualitative data) were implemented. For gene expression studies, one-way ANOVA followed by Fisher's LSD (PGC-1α ^+/+^, ^+/−^, and ^−/−^ mice) or two-tailed t-tests (R6/2 and WT mice) were implemented. For HPLC, two-tailed t-tests comparing PGC-1α ^+/+^ and ^−/−^ mice were used.

## Results

### Early onset of severe motor impairment in mice lacking PGC-1α

Adult PGC-1α ^−/−^ mice were initially reported to exhibit hindlimb clasping and hyperactivity, behavioral abnormalities reminiscent of HD, accompanied by neurodegeneration at four months of age [13]. Our own observations of home cage behavior of these mice led us to hypothesize that behavioral abnormalities occurred well before adulthood and were more severe than previously reported. To test our hypotheses, we conducted a battery of motor behavior tests in PGC-1α ^+/+^, ^+/−^, and ^−/−^ littermates at four and twelve weeks of age to determine if motor function progressively deteriorated with age.

**Figure 1:**
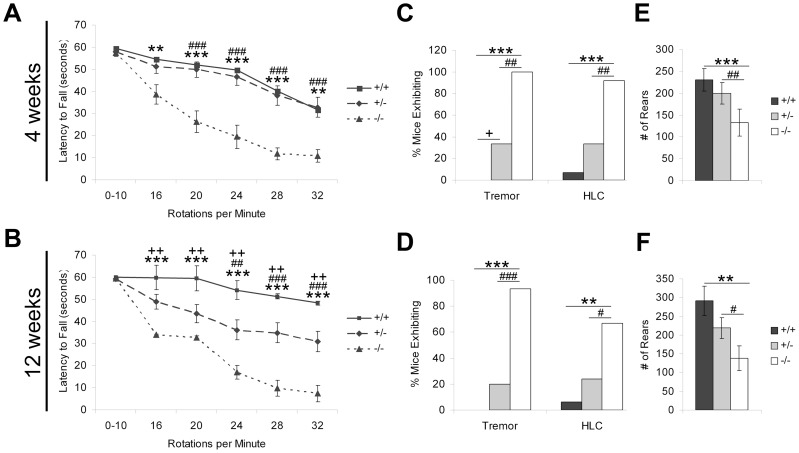
Severe motor impairment in PGC-1α ^−/−^ mice. PGC-1α ^+/+^, ^+/−^, and ^−/−^ littermates were assessed for motor coordination with rotarod analysis, for the presence or absence of tremor and hindlimb clasping (HLC), and for alterations in locomotor activity with open field monitoring at four and twelve weeks of age. **A.** At four weeks, PGC-1α ^−/−^ mice had a significantly decreased latency to fall off the rotarod apparatus compared to ^+/+^ and ^+/−^ mice at speeds of 16, 20, 24, 28, and 32 rotations per minute. **B.** By twelve weeks of age, ^+/−^, in addition to ^−/−^, mice were significantly impaired on the rotarod task compared to ^+/+^ animals. **C.** At four weeks, PGC-1α ^−/−^ and ^+/−^ mice had significantly increased instance of tremor compared to ^+/+^ mice. PGC-1α ^−/−^, but not ^+/−^, mice had increased instance of HLC. **D.** PGC-1α ^−/−^, but not ^+/−^, animals had increased instance of tremor and HLC at twelve weeks. **E–F.** PGC-1α ^−/−^ mice exhibited fewer rears in the open field paradigm compared to ^+/+^ and ^+/−^ littermates at four (E) and twelve (F) weeks of age. Other open field measures, including ambulatory distance and time, did not differ among genotypes. For A–B, repeated-measures ANOVA followed by planned comparisons with the Holm-Bonferroni correction. * ^+/+^ versus ^−/−^. + ^+/+^ versus ^+/−^. # ^+/−^ versus ^−/−^. For C–D, chi-square tests for independence. For E–F, one-way ANOVA followed by Fisher's LSD. One symbol, p<0.05. Two symbols, p<0.005. Three symbols, p<0.0005. ^+/+^ n = 17, ^+/−^ n = 25, ^−/−^ n = 15. Data presented as mean ± SEM.

To quantify motor coordination, we conducted rotarod analysis at increasing test speeds to determine the threshold at which mice lacking PGC-1α become motor impaired. Two trials were assessed at each test speed, and each trial lasted for a maximum of 60 seconds. At four weeks of age, repeated-measures ANOVA revealed a significant effect of genotype (F_(2,53)_ = 12.44, p = 3.73×10^−5^) and of rotarod speed (F_(5,265)_ = 58.83, p = 6.14×10^−41^) as well as an interaction between the two (F_(10,265)_ = 4.56, p = 5.56×10^−6^) on latency to fall. Posthoc analyses demonstrated that ^−/−^ mice had a significantly decreased latency to fall compared to ^+/+^ at 16 rotations per minute and to ^+/+^ and ^+/−^ mice at 20, 24, 28, and 32 rotations per minute (Holm-Bonferroni, p<0.005; Figure 1A). At twelve weeks of age, repeated-measures ANOVA again revealed a significant effect of genotype (F_(2,53)_ = 20.89, p = 1.94×10^−7^) and of rotarod speed (F_(5,265)_ = 58.75, p = 5.30×10^−41^) as well as an interaction between the two (F_(10,265)_ = 7.95, p = 3.48×10^−11^) on latency to fall. However, at this age, posthoc analyses revealed that both ^−/−^ and ^+/−^ mice had a significantly decreased latency to fall compared to ^+/+^ mice at 16, 20, 24, 28, and 32 rotations per minute (Holm-Bonferroni, p<0.008; Figure 1B). Interestingly, ^−/−^ mice also fell off of the rotarod significantly earlier than ^+/−^ mice at 24, 28, and 32 rotations per minute, perhaps indicating a direct relationship between expression of PGC-1α and motor performance as animals age. Of note, neither ^−/−^ nor ^+/−^ mice exhibited difficulty maintaining balance on the rotarod at 0–10 accelerating rotations per minute, suggesting that the observed effects are specific to motor coordination and not due to an inability to maintain balance on the rotarod apparatus.

While a prior report indicated that adult PGC-1α ^−/−^ animals exhibit hindlimb clasping, during our monitoring of home cage behavior, it became clear that they also exhibited a pronounced tremor. To quantify these traits, we coded for the presence or absence of tremor and hindlimb clasping at four and twelve weeks of age. At four weeks of age, ^−/−^ mice (100%) had significantly increased instance of tremor compared to ^+/+^ (0%, χ^2^ = 26.00, p = 3.41×10^−6^) and ^+/−^ (33.33%, χ^2^ = 14.64, p = 0.001) mice, and ^+/−^ mice had significantly increased instance of tremor compared to ^+/+^ mice (χ^2^ = 5.53, p = 0.02; Figure 1C). PGC-1α ^−/−^ mice (91.67%) also had significantly increased instance of hindlimb clasping compared to ^+/+^ (7.14%, χ^2^ = 18.58, p = 1.63×10^−4^) and ^+/−^ (33.33%, χ^2^ = 11.84, p = 0.006) mice. At twelve weeks of age, the results were similar. PGC-1α ^−/−^ mice (93.33%) exhibited increased instance of tremor compared to ^+/+^ (0%, χ^2^ = 27.23, p = 1.81×10^−6^) and ^+/−^ (20%, χ^2^ = 20.22, p = 6.91×10^−5^) mice (Figure 1D). As previously reported, PGC-1α ^−/−^ mice (66.67%) also had increased instance of hindlimb clasping compared to ^+/+^ (6.25%, χ^2^ = 9.57, p = 0.002) and ^+/−^ (24%, χ^2^ = 7.11, p = 0.008) mice at this age. Unlike the rotarod task, where performance of ^+/−^ worsened compared to ^+/+^ mice with age, the presence of tremor was not statistically different in ^+/−^ compared to ^+/+^ animals at twelve weeks of age.

To assess locomotor activity, mice were placed in a novel open field, and activity was tracked over a 30-minute interval with an automated system. At four and twelve weeks of age, we were unable to recapitulate the hyperactive phenotype previously described for PGC-1α ^−/−^ animals [13]. No differences were found among ^+/+^, ^+/−^, and ^−/−^ mice for ambulatory distance or time (data not shown), indicating that loss of PGC-1α does not affect general locomotor activity in this behavioral paradigm. However, at four weeks (F_(2,49)_ = 5.67, p = 0.006) and twelve weeks (F_(2,47)_ = 6.64, p = 0.003) of age, ^−/−^ mice reared on the hindlimbs significantly fewer times than ^+/+^ or ^+/−^ mice (Fisher's LSD, p<0.05; Figure 1 E–F). As the decreased rears do not correspond with decreased locomotion, this effect may be attributable to a reduced ability for ^−/−^ mice to support themselves on the hindlimbs alone rather than to a decrease in overall activity.

### Early postnatal development of spongiform vacuoles

**Figure 2:**
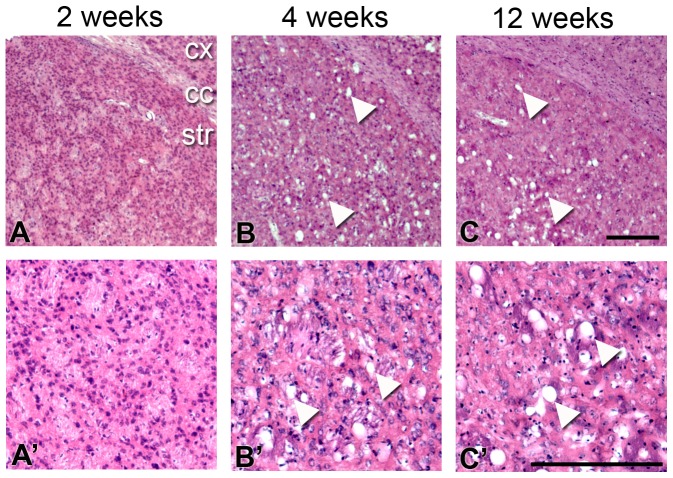
Early postnatal development of spongiform vacuolation in PGC-1α ^−/−^ striatum. Hematoxylin and eosin (H&E) stained coronal sections from PGC-1**α ^−/−^** mice at two, four, and twelve weeks demonstrate the appearance of vacuoles between two weeks (**A**; no vacuoles present) and four weeks (**B**; arrowheads) of age. Notably, vacuoles do not appear to increase in size or number between four weeks and twelve weeks (**C**) of age, indicating that neurodegeneration does not progress as animals transition from the early postnatal period to adulthood. No vacuoles were observed in age-matched PGC-1α **^+/+^** littermates (data not shown). A′–C′ are higher power images from the sections in A–C. Cx, cortex. Cc, corpus callosum. Str, striatum. Scale bars = 250 µm.

Due to the early presentation of motor symptoms in PGC-1α ^−/−^ mice, we hypothesized that striatal neurodegeneration would also be present by four weeks of age. To determine the age of onset of striatal vacuolation, we performed histological analysis with H&E staining on coronal sections from PGC-1α ^+/+^ and ^−/−^ littermates at one, two, four, and twelve weeks of age (Figure 2). At one (not shown) and two weeks (Figure 2A), no vacuoles were apparent. By four weeks of age, vacuoles were present throughout the PGC-1α ^−/−^ striatum with the greatest severity occurring in the dorsolateral region. Interestingly, vacuoles did not appear to differ in number or size between four and twelve weeks of age, indicating that striatal degeneration does not progress as animals transition from the early postnatal period to adulthood. Given our previous finding that PGC-1α is dramatically upregulated in the striatum between birth and four weeks of age [14], the appearance of vacuoles in this time frame likely reflects a requirement of PGC-1α for the maturation and/or survival of a specific cell type or types in this brain region. No vacuoles were present in PGC-1α ^+/+^ littermates at any age (data not shown).

### Conditional nervous system deletion of PGC-1α recapitulates the pathological and behavioral phenotype of PGC-1α ^−/−^ mice

As PGC-1α is highly expressed in peripheral tissues with high energy demands, such as skeletal muscle, heart, and liver, as well as the brain, it is difficult to conclude if the neurodegeneration and motor impairment observed in PGC-1α ^−/−^ mice are due to central or peripheral mechanisms. We and others [26] have shown that mice with conditional deletion of PGC-1α in calmodulin kinase II (CaMKII)-expressing cells show some evidence for striatal vacuoles, but since PGC-1α is concentrated primarily in GAD67-expressing populations [14], many of which do not express CaMKII, we sought to determine the influence of pan-neuronal PGC-1α deletion on striatal pathology and motor behavior.

**Figure 3:**
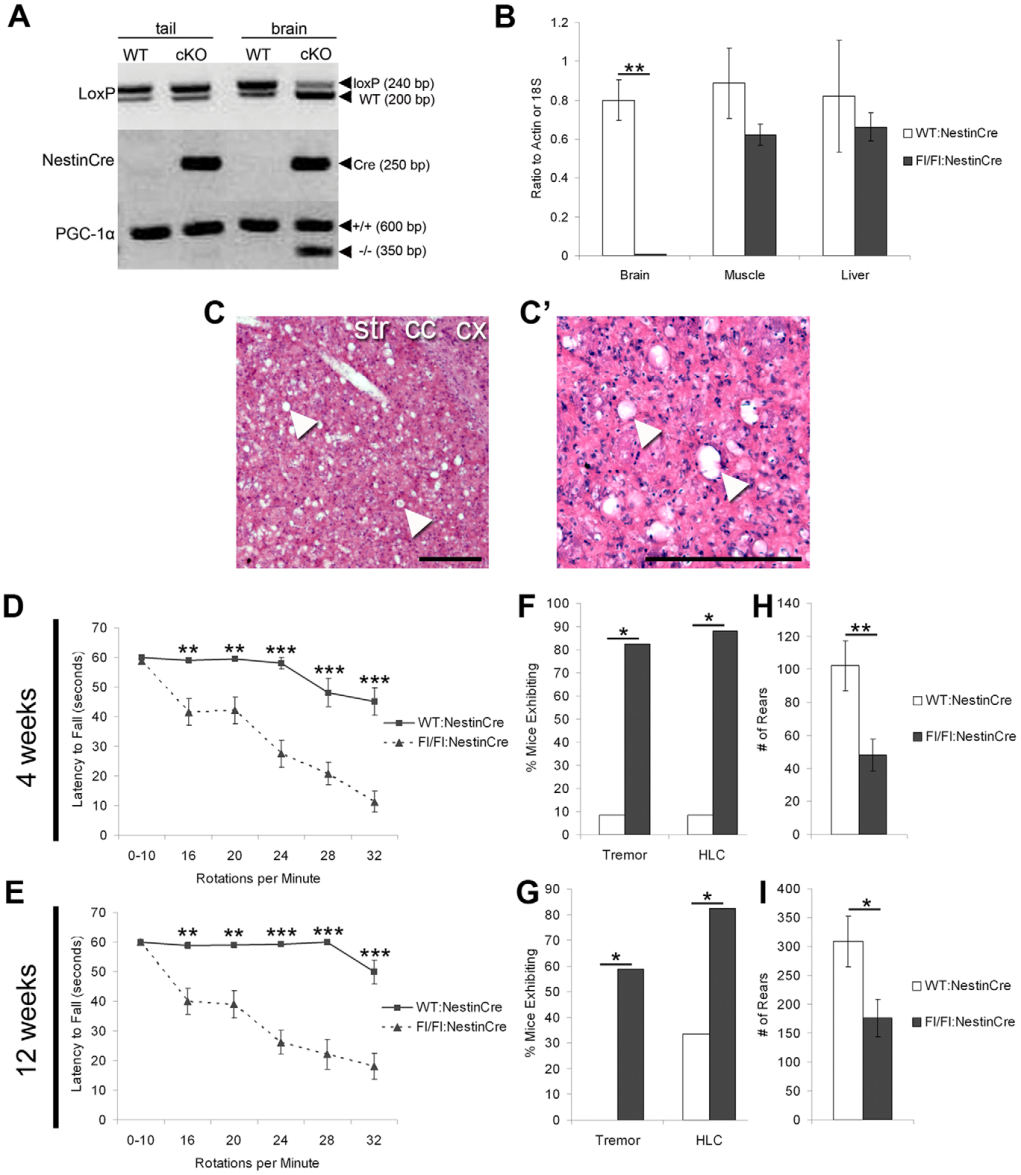
Nervous system deletion of PGC-1α recapitulates the behavioral and neurodegenerative phenotype of PGC-1α ^−/−^ mice. Nervous system conditional knockout (cKO) of PGC-1α was mediated by crossing PGC-1α^fl/fl^ mice with mice expressing cre recombinase driven by the nestin promoter. **A.** Qualitative PCR from genomic DNA from homogenized tail or brain tissue demonstrates that recombination occurs in PGC-1α^fl/+^:NestinCre brain but not tail tissue. **B.** q-RT-PCR confirmed that PGC-1α transcript expression is significantly reduced in brain but not muscle or liver of PGC-1α^fl/fl^:NestinCre compared to WT:NestinCre animals. **C.** Hematoxylin and eosin (H&E) stained coronal sections from twelve-week-old PGC-1α^fl/fl^:NestinCre mice demonstrate spongiform vacuolation in the striatum. No vacuoles were present in PGC-1α^WT^:NestinCre littermates (data not shown). Higher magnification image shown in B′. Cx, cortex. Cc, corpus callosum. Str, striatum. Scale bars = 250 µm. **D–E.** PGC-1α^fl/fl^:NestinCre mice displayed impaired motor coordination with a decreased latency to fall off the of rotarod compared to PGC-1α^WT^:NestinCre at speeds of 16–32 rotations per minute at four (C) and twelve (D) weeks of age. **F–G.** PGC-1α^fl/fl^:NestinCre exhibited increased instance of tremor and hindlimb clasping (HLC) compared to PGC-1α^WT^:NestinCre mice at four (E) and twelve (F) weeks of age. **H–I.** PGC-1α^fl/fl^:NestinCre mice reared fewer times during a 30-minute open field protocol compared to PGC-1α^WT^:NestinCre littermates at four (G) and twelve (H) weeks of age. Other behaviors of interest, including ambulatory distance and time, did not differ between genotypes at either age. D–E, repeated-measure ANOVA followed by planned comparisons with the Holm-Bonferroni correction. F–G, chi square test for independence. B;H–I, two-tailed t-tests. *p<0.05. **p<0.005. *** p<0.0005. PGC-1α^WT^:NestinCre, n = 12; PGC-1α^fl/fl^:NestinCre, n = 18. Data presented as mean ± SEM.

We produced a conditional nervous system knockout of PGC-1α by crossing mice with loxP sites flanking critical exons of the PGC-1α gene with mice expressing cre recombinase driven by the nestin promoter. To confirm specificity of cre-mediated deletion, we conducted qualitative PCR on tail and brain tissue of PGC-1α^fl/+^:NestinCre mice, which demonstrated recombination in brain but not tail tissue (Figure 3A). Moreover, PGC-1α transcript expression was significantly reduced in the brain (t _(11)_ = 7.57, p = 6.37×10^−4^) but not mixed-type muscle or liver from PGC-1α^fl/fl^:NestinCre mice (Figure 3B). H&E histological analysis on coronal sections from twelve-week-old PGC-1α^fl/fl^:NestinCre mice revealed the presence of vacuoles throughout the striatum (Figure 3C), which were comparable in occurrence and size to age-matched PGC-1α ^−/−^ mice (Figure 2C). We next assayed conditional knockouts on measures of motor functioning. Similar to PGC-1α ^−/−^ mice, PGC-1α^fl/fl^:NestinCre mice exhibited significantly impaired rotarod performance compared to PGC-1α^WT^:NestinCre mice at four weeks of age (main effect of genotype, F_(1,28)_ = 56.95, p = 3.21×10^−8^; main effect of speed, F_(5,140)_ = 24.68, p = 9.09×10^−18^; genotype x speed interaction, F_(5,140)_ = 6.62, p = 1.47×10^−5^) and twelve weeks of age (main effect of genotype, F_(1,28)_ = 57.02, p = 3.17×10^−8^; main effect of speed, F_(5,140)_ = 25.64, p = 9.52×10^−18^; genotype x speed interaction, F_(5,140)_ = 10.23, p = 1.53×10^−5^) at speeds of 16–32 rpm (Holm-Bonferroni, p<0.004; Figure 3D–E). Again, rotarod performance did not improve or decline with age. At both time points, conditional knockouts had significantly increased instance of tremor (4 weeks, 58.78%, χ^2^_(1)_ = 3.84, p = 0.05; 12 weeks, 82.35%, χ^2^_(1)_ = 6.97, p = 0.008) and hindlimb clasping (4 weeks, 82.36%, χ^2^_(1)_ = 5.92, p = 0.01; 12 weeks, 88.24%, χ^2^_(1)_ = 8.84, p = 0.003) compared to littermate controls (tremor: 4 weeks 0%, 12 weeks 8.33%; hindlimb clasping: 4 weeks 33.33%, 12 weeks 8.33%; Figure 3F–G). During a 30-minute open field monitoring session, PGC-1α^fl/fl^:NestinCre mice also exhibited fewer rears than PGC-1α^WT^:NestinCre controls (Figure 3H–I) at four (t_(28)_ = 3.27, p = 0.003) and twelve (t_(27)_ = 2.51, p = 0.02) weeks of age. As with PGC-1α ^−/−^ mice, there were no differences in other open field measurements, such as ambulatory distance and time (data not shown). These data demonstrate that peripheral mechanisms do not greatly contribute to the observed pathological and behavioral abnormalities in complete knockout animals; thus, PGC-1α ^−/−^ mice, rather than conditional knockouts, were used in the remainder of our studies.

### Transcriptional profile in the striatum of mice lacking PGC-1α and the R6/2 model of HD

Given the similarities in motor abnormalities between mice lacking PGC-1α and the well-characterized R6/2 mouse model of HD [25], we next sought to compare and contrast transcriptional markers of the basal ganglia motor pathway in these two mouse lines. Considering the primary role for PGC-1α in transcriptional regulation, we primarily concentrated our efforts on evaluating changes in transcript markers in this study. In light of the widespread vacuoles observed in PGC-1α ^−/−^ striatum and the degeneration of MSNs in R6/2 HD mice, we chose to compare their gene expression patterns specifically in the striatum. We hypothesized that if downregulation of PGC-1α expression contributes to the transcriptional alterations and striatal degeneration in HD animal models, then transcriptional changes in the striatum between these two mouse lines would be similar. Basal ganglia-mediated motor control involves two distinct pathways that play antagonistic roles: the direct pathway and the indirect pathway. In the direct pathway, MSNs project directly to the substantia nigra pars reticulata and inhibit nigral signaling. In the indirect pathway, MSNs project to the globus pallidus, disinhibiting inhibitory projections to the subthalamic nucleus and exciting nigral signaling (for an extensive review, see [27]). Locally projecting cholinergic and GABAergic interneurons of the striatum are important for the modulatory control of MSNs and are thus also involved in the control of motor behavior [28]. These distinct neuronal types express non-overlapping neuropeptides and/or dopamine (DA) receptors that allow for reliable quantification in regional homogenates. Employing a comprehensive list of transcript-specific primer/probe sets (Table 1) for q-RT-PCR, we measured gene expression of (1) general makers of MSNs, (2) direct pathway MSN markers, (3) indirect pathway MSN markers, and (4) interneuron markers in PGC-1α ^+/+^, ^+/−^, and ^−/−^ mice and R6/2 and WT mice at four and twelve weeks of age, time points that are presymptomatic and end-stage, respectively, for the R6/2 model of HD. Considering that decreases in striatum-specific transcripts are associated with cell loss and dysfunction in the R6/2 HD mouse model, we predicted that similar changes in PGC-1α ^−/−^ mice, if observed, would indicate dysfunction and/or cell loss in similar neuronal populations and pathways.

#### MSN projection markers

**Figure 4:**
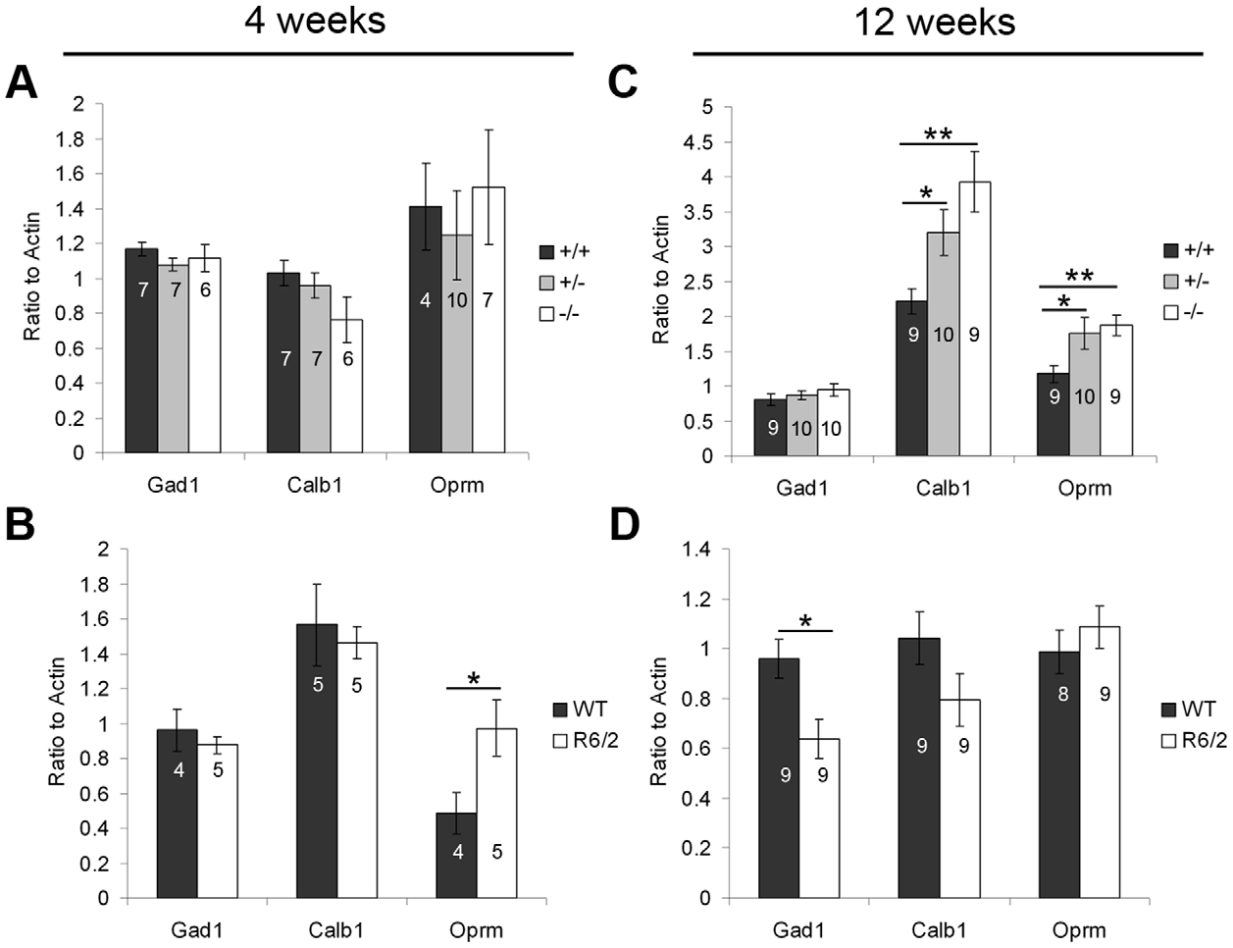
Transcriptional profile of general striatal projection markers. Gene expression of Gad67 (Gad1), calbindin (Calb1), and μ opioid receptor (Oprm) was measured in striatum of PGC-1α and R6/2 HD mice and littermate controls at four and twelve weeks using q-RT-PCR. No changes were found in mice lacking PGC-1α (**A**), but the striosome marker Oprm was significantly increased in R6/2 compared to WT mice (**B**) at four weeks of age. By twelve weeks of age, the matrix marker Calb1 and the striosome marker Oprm were significantly increased in PGC-1α ^+/−^ and ^−/−^ compared to ^+/+^ mice (**C**), and Gad1 was significantly decreased in R6/2 HD mice (**D**). For A and C, one-way ANOVA followed by Fisher's LSD. For B and D, two-tailed t-tests. *p<0.05. **p<0.005. n/group indicated on histograms. Data presented as mean ± SEM.

MSNs comprise 90–95% of the striatal neuron population and release GABA as their neurotransmitter. The Ca^2+^-binding protein calbindin (Calb1) and the μ opioid receptor (Oprm) are expressed in MSNs of both the direct and indirect pathways. Calb1 is exclusive to the matrix compartment of the striatum, while Oprm is preferentially expressed in the patch/striosome compartment [29]. We measured glutamic acid decarboxylase of 67 kDa (Gad1; the enzyme responsible for the conversion of glutamate to GABA), Calb1, and Oprm in the striatum of PGC-1α ^−/−^ and R6/2 HD mice at four and twelve weeks of age (Figure 4). Interestingly, at four weeks of age, Oprm was significantly increased in R6/2 compared to WT mice (t_(7)_ = 2.63, p = 0.03; Figure 4B), perhaps indicative of specific developmental alterations in striosomes, while no changes were observed in mice lacking PGC-1α. At twelve weeks of age, Calb1 (F_(2,27)_ = 6.11, p = 0.007) and Oprm (F_(2,28)_ = 4.64, p = 0.02) were significantly increased in ^−/−^ and ^+/−^ compared to ^+/+^ mice (Fisher's LSD, p<0.05; Figure 4C). Gad1 was significantly decreased in R6/2 compared to WT mice (t_(16)_ = 2.54, p = 0.02; Figure 4D) while Oprm was no longer increased at this age. The lack of change in Gad1 in PGC-1α ^−/−^ mice at either age suggests that there is not an overt loss of MSNs, as seen in the R6/2 mice.

#### Direct pathway markers

**Figure 5:**
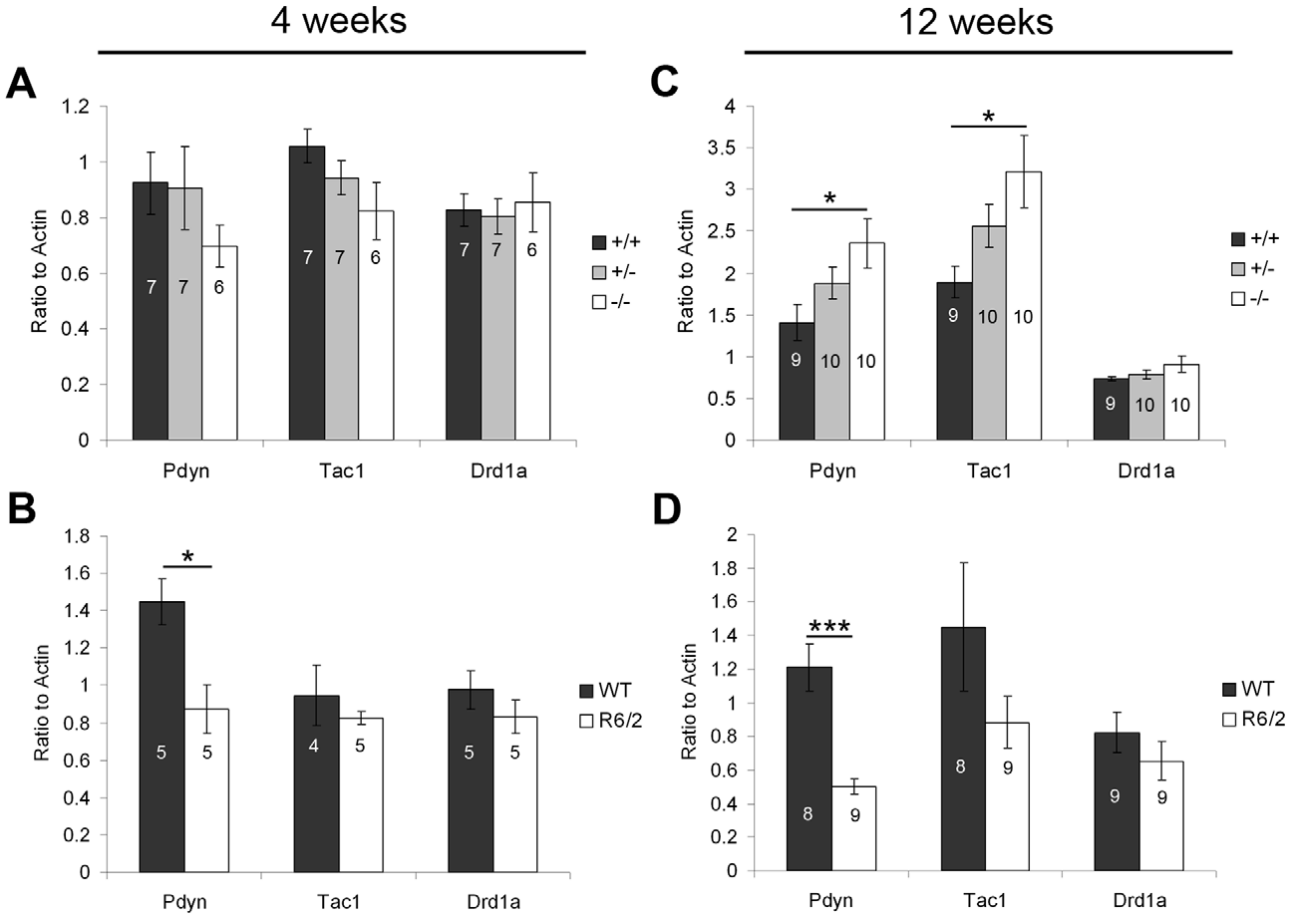
Transcriptional profile of direct pathway markers. Gene expression of prodynorphin (Pdyn), substance P (Tac1), and dopamine receptor 1a (Drd1a) was measured in striatum of PGC-1α and R6/2 HD mice and littermate controls at four and twelve weeks using q-RT-PCR. **A.** At four weeks of age, no changes in direct pathway transcripts were observed in PGC-1α ^−/−^ mice. **B.** A significant decrease in Pdyn was observed in R6/2 striatum at this age. **C.** By twelve weeks of age, expression of Pdyn and Tac1 were significantly increased in PGC-1α ^−/−^ compared to ^+/+^ mice. **D.** Pdyn was still significantly decreased in R6/2 HD mice at this age. For A and C, one-way ANOVA followed by Fisher's LSD. For B and D, two-tailed t-tests. *p<0.05. n/group indicated on histograms. Data presented as mean ± SEM.

MSNs of the direct pathway express dynorphin (Pdyn), substance P (Tac1), and DA receptor 1a (Drd1a; [30], [31]). At four weeks of age, no changes were observed in direct pathway markers in mice lacking PGC-1α (Figure 5A). A significant decrease was observed in the expression of Pdyn in R6/2 striatum at this age (t_(8)_ = 3.59, p = 0.007; Figure 5B). By twelve weeks of age, Pdyn (F_(2,28)_, = 4.48, p = 0.02) and Tac1 (F_(2,28)_ = 4.48, p = 0.02) were significantly increased in PGC-1α ^−/−^ compared to ^+/+^ mice (Fisher's LSD, p<0.05; Figure 5C). Decreased expression of Pdyn (t_(15)_ = 5.08, p = 0.0001) was still the only change observed in direct pathway markers in end-stage R6/2 HD mice (Figure 5D). Like the general MSN markers, expression of direct pathway markers increased in mice lacking PGC-1α and decreased in R6/2 HD mice with age.

#### Indirect pathway markers

**Figure 6:**
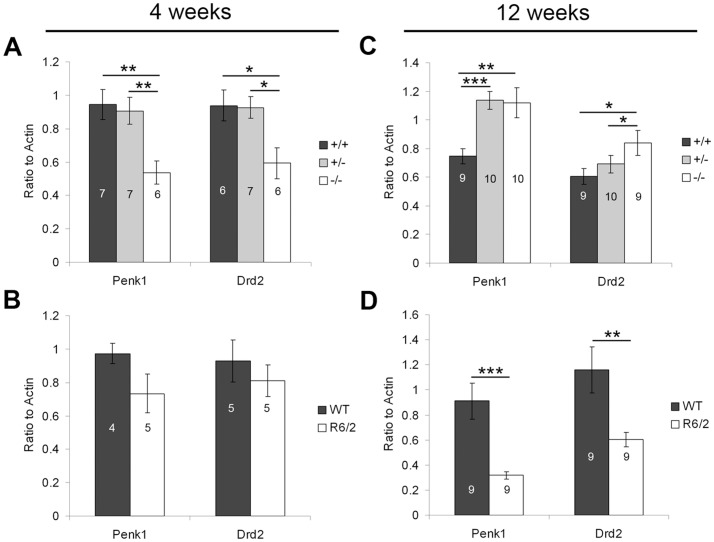
Transcriptional profile of indirect pathway markers. Gene expression of preproenkephalin (Penk1) and dopamine receptor 2 (Drd2) was measured in striatum of PGC-1α and R6/2 HD mice and littermate controls at four and twelve weeks using q-RT-PCR. **A.** At four weeks of age, expression of Penk1 and Drd2 was significantly decreased in PGC-1α ^−/−^ compared to ^+/+^ and ^+/−^ mice. **B.** No changes were observed in R6/2 HD mice at this age. **C.** By twelve weeks of age, Penk1 was significantly increased in PGC-1α ^−/−^ and ^+/−^ compared to ^+/+^ mice, and Drd2 was significantly increased in PGC-1α ^−/−^ compared to ^+/+^ and ^+/−^ mice. **D.** Both Penk1 and Drd2 were significantly decreased in R6/2 HD mice at this age. For A and C, one-way ANOVA followed by Fisher's LSD. For B and D, two-tailed t-tests. *p<0.05. **p<0.005. ***p<0.0005. n/group indicated on histograms. Data presented as mean ± SEM.

MSNs of the indirect pathway express enkephalin (Penk1) and DA receptor 2 (Drd2; [32], [33]). At four weeks of age, significant decreases in Penk1 (F_(2,19)_ = 8.60, p = 0.0003) and Drd2 (F_(2,18)_ = 5.63, p = 0.01) were observed in PGC-1α ^−/−^ compared to ^+/+^ and ^+/−^ mice (Fisher's LSD, p<0.05; Figure 6A), while no changes were observed in indirect pathway markers in R6/2 mice at this age (Figure 6B). By twelve weeks, expression of Penk1 (F_(2,28)_ = 8.32, = 0.002) and Drd2 (F_(2,27)_ = 5.33, p = 0.01) had reversed to increased expression in ^−/−^ compared to ^+/+^ and ^+/−^ mice (Fisher's LSD, p<0.05; Figure 6C). Again, this effect was in opposition to the effect in R6/2 mice, which exhibited significant decreases in Penk1 (t_(16)_ = 4.30, p = 0.0005) and Drd2 (t_(16)_ = 3.08, p = 0.007) compared to WT littermates at this age.

#### Interneuron markers

PGC-1α was previously reported to be primarily concentrated in neurons expressing high levels of GAD67 [14]. GABAergic interneurons comprise 5–10% of total neurons in the striatum and express the highest levels of GAD67, while MSNs have more moderate expression of this enzyme [34]. GABAergic interneuron subtypes in striatum can be differentiated by their expression of the Ca^2+^-binding proteins parvalbumin (Pvalb) and calretinin (Calb2), the peptide neuropeptide Y (NPY), and the enzyme neuronal nitric oxide synthase (Nos1; [35]), and these markers are expressed in largely non-overlapping populations [36]. Cholinergic interneurons expressing choline acetyltranferase (Chat) represent the other major interneuron population in this brain region and were also included for analysis.

**Figure 7:**
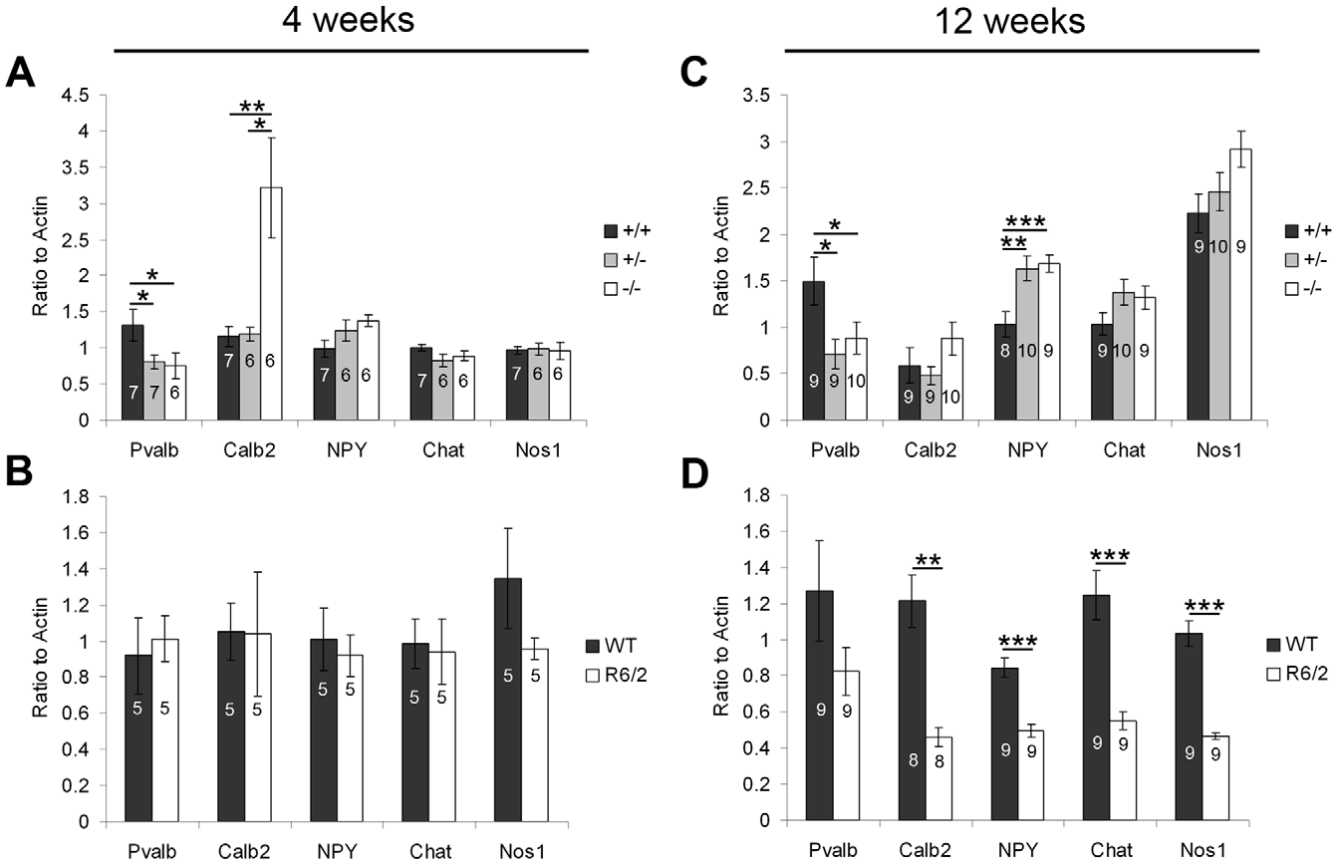
Transcriptional profile of interneuron markers. Gene expression of parvalbumin (Pvalb), calretinin (Calb2), neuropeptide Y (NPY), choline acetyltransferase (Chat), and neuronal nitric oxide synthase (Nos1) was measured in striatum of PGC-1α and R6/2 HD mice and littermate controls at four and twelve weeks using q-RT-PCR. **A.** At four weeks of age, Pvalb was significantly decreased in PGC-1α ^−/−^ and ^+/−^ compared to ^+/+^ mice, and Calb2 was significantly increased in PGC-1α ^−/−^ compared to ^+/+^ and ^+/−^ mice. **B.** No changes in interneuron markers were observed in R6/2 HD mice at this age. **C.** At twelve weeks of age, Pvalb was still significantly decreased in PGC-1α ^−/−^ and ^+/−^ compared to ^+/+^ mice, and expression of NPY was significantly increased in ^−/−^ and ^+/−^ compared to ^+/+^ mice. **D.** In the R6/2, all interneuron markers except Pvalb were significantly decreased at this age. For A and C, one-way ANOVA followed by Fisher's LSD. For B and D, two-tailed t-tests. *p<0.05. **p<0.005. ***p<0.0005. n/group indicated on histograms. Data presented as mean ± SEM.

At four weeks of age, a significant reduction in Pvalb expression was found in PGC-1α ^−/−^ and ^+/−^ compared to ^+/+^ mice (F_(2,19)_ = 5.28, p = 0.02; Fisher's LSD, p<0.05), as previously reported [37]. There was also a significant increase in the expression of Calb2 at this age (F_(2,18)_ = 10.58, p = 0.001; Fisher's LSD, p<0.05; Figure 7A). No changes in interneuron markers were observed in presymptomatic R6/2 mice (Figure 7B). At twelve weeks of age, Pvalb (F_(2,27)_ = 4.73, p = 0.02) was still significantly reduced in ^−/−^ and ^+/−^ compared to ^+/+^ mice, Calb2 was no longer increased, and NPY (F_(2,26)_ = 8.92, p = 0.001) was increased in ^−/−^ and ^+/−^ compared to ^+/+^ mice (Fisher's LSD, p<0.05; Figure 7C). In the R6/2, expression of Calb2 (t_(9)_ = 4.31, p = 0.002), NPY (t_(14)_ = 5.85, p = 4.21×10^−5^), Nos1 (t_(9)_ = 8.17, p = 1.86×10^−5^), and Chat (t_(10)_ = 5.15, p = 0.0004) was significantly decreased compared WT mice at this age (Figure 7D). Of note, the only striatal marker found to be decreased in PGC-1α ^−/−^ mice at twelve weeks of age (Pvalb) was one of the few markers not decreased in end-stage R6/2 HD mice.

### Confirmation of selected transcript changes at the protein level

**Figure 8:**
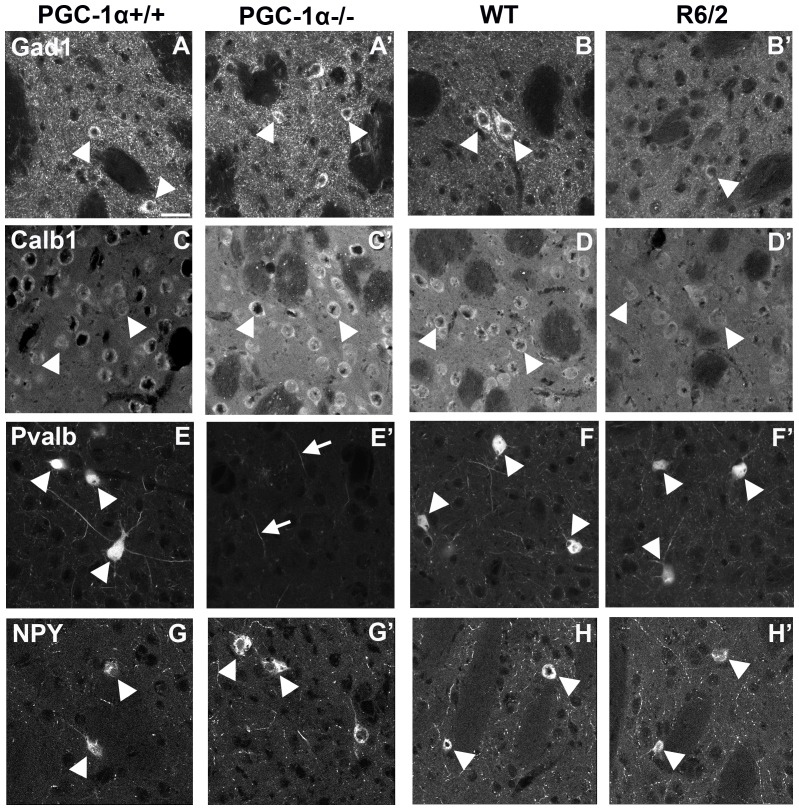
Evaluation of selected medium spiny neuron (MSN) and interneuron markers in the striatum of PGC-1α −/− and R6/2 mice. Representative confocal images of coronal sections of the striatum stained with antibodies directed against Gad1 (A–B), Calb1 (C–D), Pvalb (E–F), and NPY (G–H) demonstrate that protein expression changes closely mirror those at the transcript level in PGC-1α ^+/+^ and ^−/−^ and R6/2 and WT littermates. PGC-1α ^−/−^ mice exhibit no change in Gad1 expression (A′, arrowheads), while Calb1 (C′) and NPY (G′) expression was increased in cell bodies (arrowheads) and processes of PGC-1α ^−/−^ mice compared to ^+/+^ mice (A,C,G). Pvalb was minimally expressed in PGC-1α ^−/−^ cell bodies (E′); however, some staining was still evident in processes (arrows). Notably, overall MSN and interneuron number did not appear to differ between PGC-1α ^+/+^ and ^−/−^ mice. Expression of Gad1 (B′), Calb1 (D′), and NPY (H′) were reduced in R6/2 versus WT animals (B,D,H), while expression Pvalb remained unaltered (D–D′). Decreases in Gad1 and Calb1 appeared to reflect a loss of expressing cells as well as decreased expression in remaining cells (arrows), while decreases in NPY appeared solely to reflect a loss of expression within this cellular population (arrows) in the R6/2. Scale bar = 25 µm.

To determine if transcript expression changes observed in the PGC-1α ^−/−^ and R6/2 mouse lines reflected changes at the protein level, we conducted immunofluorescence staining on coronal sections of the striatum from both lines at twelve weeks of age. We probed for the MSN projection marker Calb1, the interneuron markers Pvalb and NPY, and the MSN and interneuron co-marker Gad1. Histological data were highly in accordance with the q-RT-PCR data for both mouse lines (Figure 8). PGC-1α ^+/+^ (A) and ^−/−^ (A′) striatum exhibited similar Gad1 fluorescence intensity in cell bodies and puncta of axonal projections (Figure 8), suggesting that PGC-1α is not crucial to the viability of striatal inhibitory neuron populations. In accordance with this view, we observed greatly increased Calb1 staining in the matrix compartment of the striatum in PGC-1α ^−/−^ (C′) compared to ^+/+^ (C) animals, despite the observation that similar numbers of MSNs appeared to be labeled with Calb1 in ^+/+^ and ^−/−^ mice. Very few Pvalb-labeled interneurons were observed in PGC-1α ^−/−^ striatum (E′), consistent with Pvalb being a PGC-1α-dependent gene; however, Pvalb-positive processes were still apparent. While the number of NPY-positive interneurons appeared to be similar between PGC-1α ^−/−^ (G′) and ^+/+^ (G) mice, a higher intensity of NPY fluorescence was observed in ^−/−^ cells and processes.

R6/2 mice demonstrated significant loss of Gad1 (B′), Calb1 (D′), and NPY (H′) staining in the striatum compared to WT littermates (B,D,H). In the case of Gad1 and Calb1, fewer cells appeared to be stained, and the remaining cells exhibited decreased intensity of staining. NPY cell number did not appear to be greatly affected in the R6/2 (H′); however, labeled cells exhibited decreased staining intensity compared to those in the WT animals (H). Despite alterations in both MSN and interneuron markers in the R6/2 striatum, Pvalb expression remained unchanged between WT (F) and R6/2 (F′) animals.

### No change in PGC-1α transcript expression in R6/2 striatum

Given the striking opposition in gene expression of basal ganglia motor pathway markers in the striatum of PGC-1α ^−/−^ and R6/2 HD mice, we sought to replicate the previous report of decreased PGC-1α transcript expression in this particular model of HD [23]. We measured gene expression of the functionally-required portion of the PGC-1α transcript (exon 5–6) and found no significant decreases in R6/2 (4 weeks, 0.87±0.13 a.u.; 12 weeks, 0.90±0.07 a.u.) compared to WT (4 weeks, 0.86±0.20 a.u.; 12 weeks, 0.75±0.09 a.u.) striatum at either age tested (two-tailed t-tests, p>0.05). We have previously demonstrated the efficacy of this primer probe set in PGC-1α ^−/−^ hippocampus [37]; in the striatum, minimal PGC-1α expression existed in ^−/−^ mice (0.02±0.02 a.u.), while ^+/−^ mice (0.80±0.07 a.u.) had a ∼50% reduction from ^+/+^ mice (1.52±0.22 a.u.). We then measured additional PGC-1α transcripts (exon regions 1–2 and 10–11) in the R6/2 striatum at four and twelve weeks of age; no changes were found in either of these transcripts at either age (data not shown). The lack of replication of the previous reports of decreased PGC-1α in the striatum of R6/2 is not surprising, as decreases in this and other mouse models of HD [16], [22] have been extremely modest (∼10% change).

### Striatal dopamine levels are not altered in PGC-1α ^−/−^ mice

Considering changes in DA receptor expression and the emerging role of PGC-1α in Parkinson disease [38], [39], we investigated whether total DA levels were altered in the striatum, potentially contributing to the observed changes in gene expression and motor coordination. Altered DA signaling to MSNs from the substantia nigra and ventral tegmental area has been shown to differentially effect direct and indirect pathway MSN gene expression. Of the five DA receptor subtypes, Drd1a and Drd2 are the most enriched in the striatum; and, as previously mentioned, direct pathway MSNs preferentially express Drd1a, while indirect pathway MSNs preferentially express Drd2 [30]–[32]. Both Drd1a and Drd2 are G-protein coupled receptors, and activation of Drd1a stimulates, while activation of Drd2 inhibits, adenylyl cyclase activity and corresponding downstream signaling cascades [40]. It has been shown that activation of Drd1a increases gene expression of Pdyn and Tac1, while activation of Drd2 reduces gene expression of Penk1 [30], [41]. Thus, increased levels of DA could account for the increased transcript expression of Pdyn and Tac1 in PGC-1α ^−/−^ mice at 12 weeks. Conversely, decreased levels of DA could account for the increased expression of Penk1 in PGC-1α ^−/−^ and ^+/−^ mice at this age.

**Figure 9:**
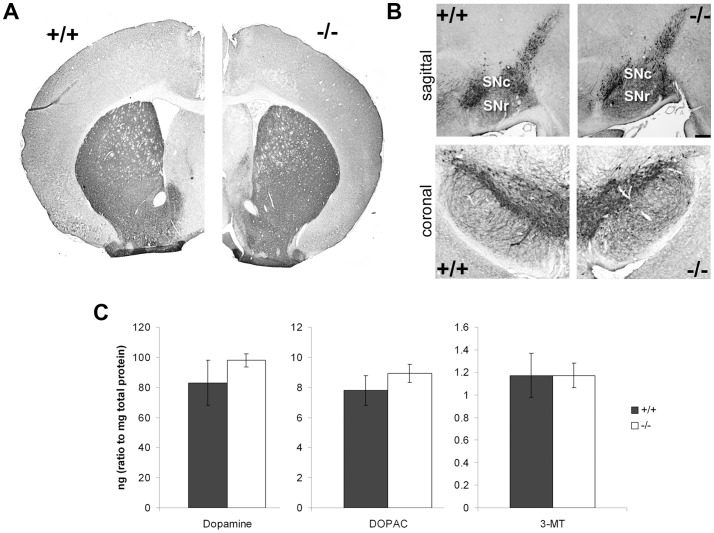
Loss of PGC-1α does not alter tyrosine hydroxylase or dopamine levels. Immunohistochemistry for tyrosine hydroxylase (TH) and high performance liquid chromatography (HPLC) for dopamine and its metabolites were used as measures of dopamine in PGC-1α ^+/+^ and ^−/−^ mice at twelve weeks of age. **A–B.** Representative low magnification pictures of immunohistochemistry with an antibody directed against TH revealed no differences in the distribution of immunoreactivity of axon fibers to the striatum (A) or cell bodies in the substantia nigra (B) in PGC-1α ^−/−^ mice. SNc, substantia nigra pars compacta; SNr, substantia nigra pars reticulata. Scale bars = 1 mm. **C.** Quantification of HPLC probing biogenic amines demonstrated that neither dopamine nor its metabolites (DOPAC and MT-3) are significantly altered in −/− mice. +/+, n = 4; −/−, n = 7. Data presented as mean ± SEM.

To determine if altered DA signaling could contribute to the transcriptional changes observed in mice lacking PGC-1α ^−/−^, we initially conducted immunohistochemistry to determine the integrity of dopaminergic brain regions. Using an antibody against tyrosine hydroxylase (TH), the enzyme responsible for conversion of tyrosine to the DA precursor DOPA, we visualized the substantia nigra and ventral tegmental area, as well as their axon projections to the striatum, in PGC-1α ^+/+^ and ^−/−^ mice at twelve weeks of age. No differences were observed in the cellular distribution of TH staining in axon fibers in the striatum (Figure 9A) or cell bodies in the substantia nigra (Figure 9B) and ventral tegmental area (data not shown) in ^+/+^ versus ^−/−^ mice, indicating that dopaminergic brain regions are intact in PGC-1α ^−/−^ mice. However, as TH is an indirect measure of DA, we also conducted HPLC to measure biogenic amines in striata from microwave-fixated brains from PGC-1α ^+/+^ and ^−/−^ mice at twelve weeks of age. Consistent with the TH staining results, no differences were found in total levels of DA or its metabolites (DOPAC and MT-3) in ^−/−^ compared to ^+/+^ animals (Figure 9C), suggesting that a lack of PGC-1α alone is not sufficient to cause overt loss of dopaminergic neuron viability at this age.

## Discussion

An accumulating body of evidence strongly implicates the transcriptional coactivator PGC-1α in the pathology of HD. Here, we report for the first time that PGC-1α ^−/−^ mice exhibit severe rotarod deficits, tremor, and decreased rearing behavior, in addition to the previously described hindlimb clasping, as early as four weeks of age. These behavioral changes were accompanied by the appearance of spongiform vacuoles between the second and fourth postnatal week; however, vacuoles did not appear to be attributable to overt MSN cell loss. While these behavioral deficits mimic those observed in the R6/2 mouse model of HD, the R6/2 mouse generally does not exhibit a behavioral phenotype until eight weeks of age, at which point motor impairment progressively worsens until premature death at 12–14 weeks [24], [25]. Rather unexpectedly, PGC-1α ^−/−^ mice did not exhibit a progressive deterioration of motor function between four and twelve weeks but rather presented similar magnitude of effect on rotarod performance, rearing behavior, and the presence of hindlimb clasping, tremor, and spongiform vacuoles at both ages. PGC-1α ^+/−^ mice, on the other hand, demonstrated a progressive decline of rotarod performance compared to ^+/+^ mice between four and twelve weeks and, therefore, may be a better model of progressive motor deterioration occurring in HD and other neurological disorders than the ^−/−^ animal.

By directly comparing the expression of genes of the basal ganglia motor circuit in the striatum of mice lacking PGC-1α with R6/2 HD mice, it is clear that the transcriptional abnormalities between these two mouse models do not mirror but directly oppose one another. In humans, HD is first characterized by involuntary movements (chorea) followed by an inability to execute motor actions (bradykinesia). Basal ganglia wiring diagrams suggest that this progression of symptoms is mediated by dysfunction of the indirect pathway followed by dysfunction of the direct pathway [42], [43]. However, more recent empirical data in mice suggest that activation of the direct pathway increases, while activation of the indirect pathway decreases, locomotor behavior [44], perhaps indicating a difference between motor pathways in humans and rodents. Our data suggest that, in the R6/2 mouse, the direct pathway is affected before the indirect pathway, as Pdyn was significantly decreased in the presymptomatic R6/2 HD striatum at four weeks of age. Increased expression of the striosome marker Oprm, which has been reported to be more highly localized to direct rather than indirect pathway MSNs [45], was the only other transcriptional change in R6/2 HD mice at this age. However, it is unclear whether the observed change of Oprm is the result of increased expression in direct pathway MSNs, indirect pathway MSNs, or both. While the literature generally supports that the indirect pathway is affected first in the R6/2, these prior reports have been limited by consistently using Tac1, rather than Pdyn, as the only direct pathway maker [46]–[49]. In contrast, mice lacking PGC-1α have significantly decreased expression of indirect pathway markers (Penk1 and Drd2) and the interneuron marker Pvalb at this age.

By end-stage, almost all tested markers, including Gad1, Pdyn, Penk1, Drd2, Calb2, NPY, Nos1, and Chat, were significantly decreased in the R6/2 striatum. These findings are largely consistent with prior investigations of this brain region in R6/2 transgenic mice [50]–[53]. The gene expression changes that we and others have observed in end-stage R6/2 striatum, such as decreased expression of Penk1, Drd2, Pdyn, NPY, and Nos1 and no change in Oprm or Pvalb, exhibit parallels with postmortem human HD caudate samples [54], [55]. However, human HD samples also have dramatically decreased transcript expression of Calb1 and Tac1 [54], [55], which we did not observe at any time point in the R6/2. While gross neuronal loss was not initially reported in these animals [24], significantly decreased striatal volume and neuron number accompanied by increased ventricle size and histological changes indicative of degenerative processes are apparent in R6/2 HD mice by twelve weeks of age [56], [57]. Thus, it is likely that the large transcriptional changes that we and others have observed in the end-stage R6/2 are due to loss of cellular viability in this brain region.

Mice lacking PGC-1α, on the other hand, showed increased expression of striosome (Oprm), matrix (Calb1), direct pathway (Pdyn, Tac1), indirect pathway (Penk1, Drd2), and interneuron (NPY) markers at twelve weeks of age, while the previously published PGC-1α-dependent gene Pvalb [37] was the only transcript still decreased at this age. These data are in direct contrast to the gene expression changes found in the R6/2 striatum and postmortem human HD caudate tissue samples. Furthermore, these increases are remarkable considering the spongiform vacuoles present in the striatum of PGC-1α ^−/−^ mice (Figure 2; [13]). The transcript data together with the histology data demonstrating similar numbers of Calb1- and Gad1-positive cells indicate that MSNs are not lost (at least not to a great extent) in these animals. In support of this idea, even at six months of age, ventricle size and striatal volume do not appear to differ between PGC-1α ^−/−^ mice and littermate controls (unpublished observation, EKL; also see Figure 9), as does in the R6/2. The lack of correlation between changes in mice lacking PGC-1α and R6/2 HD mice are not entirely surprising given that we were unable to replicate a previous report of decreased expression of PGC-1α in R6/2 striatum [23], a result further validated by a lack of change in transcript expression of the PGC-1α-dependent gene Pvalb. However, cellular specificity may be a key element in accurately measuring changes in PGC-1α, as cell-specific gene expression analyses have shown a decrease of PGC-1α in MSNs (∼5 fold) and an increase in Nos1-positive interneurons (∼50 fold) in an HD mouse model [15].

As a transcriptional coactivator, PGC-1α functions to initiate gene transcription. Therefore, loss of PGC-1α should cause a decrease, rather than an increase, in target gene expression. Considering the data presented here, we hypothesize that significantly increased transcripts in PGC-1α ^−/−^ striatum are not transcriptional targets of PGC-1α and that these changes are not directly attributable to loss of PGC-1α alone. It is possible that the observed increases in MSN markers reflect a compensatory change induced by loss of unidentified PGC-1α-dependent genes in MSNs or other cellular populations. As PGC-1α has been implicated in the control of metabolic genes in the brain [13], [58], it is possible that reductions in antioxidant defense or mitochondrial function lead to widespread transcriptional alterations. The dramatic upregulation of transcript expression in ^−/−^ mice between four and twelve weeks also raises the possibility that PGC-1α controls the expression of a generic repressor, that when lacking, allows for the upregulation of these transcripts. This study also highlights the idea that a preferable age to look for proximal effects, as opposed to compensatory mechanisms, in PGC-1α ^−/−^ mice is ≤4 weeks of age when few changes are present in PGC-1α-independent genes but changes in previously established PGC-1α-dependent genes (i.e. Pvalb; [37]) are detectable. It also would be informative to investigate the impact of PGC-1α deletion in basal ganglia motor circuits in adulthood to determine whether loss of PGC-1α from a developmentally normal motor circuit would be sufficient to cause similar motor deficits, spongiform vacoulation, and compensatory increases in MSN transcripts.

In an attempt to explain the increases in direct and indirect pathway markers at a transcriptional level, we measured DA content in PGC-1α ^−/−^ animals, as increased/decreased levels of DA would explain the increased expression of direct/indirect pathway markers, respectively [30], [41]. TH immunoreactivity in ^−/−^ mice demonstrated that dopaminergic brain regions (substantia nigra and ventral tegmental area) as well as their axon terminals in the striatum were intact and expressed levels of TH comparable to ^+/+^ littermates, consistent with a prior investigation of TH in six-week-old animals [58]. HPLC analyses confirmed that the expression of DA and its metabolites DOPAC and MT-3 were not significantly altered in ^−/−^ compared to ^+/+^ mice. While this data does not offer an explanation for the transcriptional abnormalities observed in ^−/−^ mice, it suggests that nigral dopaminergic neurons are intact. However, it remains to be seen whether DA release from terminals is affected; microdialysis experiments would be necessary to investigate this possibility.

Recent publications have implicated PGC-1α in the pathology of Parkinson disease [38], [39], a disorder also characterized by motor dysfunction, mitochondrial abnormalities, and oxidative stress. Behaviorally, PGC-1α ^−/−^ mice exhibit similarities with animal models of Parkinson disease [59] but do not display the dopaminergic cell death characteristic of the disease. However, when considering our developmental observations, it is important to note that a gradual loss of PGC-1α in adulthood may elicit different transcriptional changes than a lack of PGC-1α from conception, as is the case for PGC-1α ^−/−^ mice, or during neurogenesis, as is the case for PGC-1α ^fl/fl^:NestinCre mice. Motor deficits are quantifiable as early as four weeks after birth in these mice, suggesting that PGC-1α is required for the proper development and maintenance of motor circuitry. After this initial requirement is fulfilled, PGC-1α's role in the brain may shift to one of neuroprotection. While loss of PGC-1α itself does not alter DA content, loss of PGC-1α exacerbates [58] while overexpression of PGC-1α protects against [60] dopaminergic cell death in the MPTP model of Parkinson disease.

From this study, it is still unclear why loss of PGC-1α results in striatal degeneration similar to that seen in spongiform encephalopathies such as prion disease but without additional degeneration following the initial onset between the second and fourth week of postnatal life. The appearance of vacuoles occurs at a time of dramatic upregulation of PGC-1α expression in this brain region [14], suggesting that PGC-1α is required for the proper development and/or survival of the cellular population undergoing degeneration. The transcriptional compensation of MSNs suggests that the observed vacuoles are not due to loss of main projection cells within the striatum but does not rule out the possibility that these neurons undergo a loss of cellular mass. A recent publication demonstrated that conditional deletion of PGC-1α by CamKII, an enzyme preferentially expressed in forebrain projection neurons, but not interneurons or glia, recapitulates the striatal vacuolation neurodegenerative phenotype of PGC-1α ^−/−^ mice [26]. However, as PGC-1α ^−/−^ mice have also been found to have deficits in myelination [61], it is possible that local demyelination and/or developmental delays in myelination could result in deafferentation of the striatum and compensatory mechanisms in MSNs. Thus, it is difficult to conclude which cellular populations are contributing to the vacuolation. Experiments involving cell-specific deletion of PGC-1α are required to resolve this question.
